# Supplementary material for: Are carbon emissions trading and green financial instruments synergistic? -Comprehensive quantitative research based on content analysis
Source: PLoS One. 2024 Mar 7;19(3):e0298601. doi: 10.1371/journal.pone.0298601 (PMC10919601; doi:10.1371/journal.pone.0298601)
Supplement: S1 Data — (ZIP) [file pone.0298601.s001.zip › Data/地级市Z_F工作报告.zip!/║┌┴·╜¡/╞▀╠¿║╙╩╨/└┤╫╘╣1⁄2╓┌║┼í╛┬φ┐╦╩2╛▌═°í┐_2018.docx]

各位代表：

　　现在，我代表市人民政府，向大会报告工作，请予审议，并请政协委员和列席人员提出意见。

　　一、2017年工作回顾

　　2017 年，面对复杂多变的宏观经济环境，我们在以习近平同志为核心的党中央坚强领导下，认真贯彻落实党中央国务院、省委省政府和市委各项决策部署，围绕“激发内生动力，加快转型振兴”目标，自觉践行新发展理念，坚持稳中求进工作总基调，定向施策，精准发力，统筹做好改革发展稳定各项工作，较好地完成了市十一届人大一次会议确定的各项目标任务，经济回暖企稳，社会和谐稳定。初步预计，全市生产总值实现230亿元，增长6.0%，增速为五年来最高；规模以上工业增加值实现65 亿元，增长4.5%；财政一般公共预算收入实现21.9亿元，增长37.0% ；城镇居民人均可支配收入实现23506元，增长6.5%；农村居民人均可支配收入实现12112 元，增长6.2%。

　　大力推进产业项目建设，新发展动能加速孕育。围绕做好“三篇大文章”，创新招商引资和项目推进机制，大力发展六大产业，推进千万元以上产业项目45 个，累计完成投资97.4 亿元。贯彻落实“煤头化尾”，大力发展现代煤化工产业，宝泰隆30 万吨稳定轻烃、隆鹏20 万吨清洁化学品、勃盛焦化升级改造等煤化工大项目顺利推进，即将全面建成投产。加快转化新材料产业科技成果，鑫科纳米1 万吨石墨烯防腐涂料、3000 吨石墨烯钛纳米聚合物涂料项目建成试产，万锂泰5 万吨锂电池负极材料产业集群项目、鑫科纳米2 万吨耐高温石墨烯树脂项目开工建设。贯彻落实“农头工尾”，大力发展生物发酵产业，中瑯生物5 万吨衣康酸10 万吨葡萄糖酸钠项目开工建设，兴粮30 万吨玉米深加工项目实现试产。贯彻落实“粮头食尾”，大力发展绿色食品产业，金穗米业30 万吨稻谷综合加工、天泰食品项目建成投产。坚持用先进技术改造提升制造业，实施双叶家具生产线智能改造升级，晟斯凯特铁路专用岔枕项目建成投产，升华医疗器械项目加快推进。积极引入新业态、新商业模式，中国供销商贸博览城一期投入运营，海生跨境电商新零售小镇项目签约落地。全面推动“双创”，电商及相关企业达160 户，线上交易额突破3 亿元，吸纳就业超过7000 人；认定“创业孵化基地”10 家，入驻企业200 余家，带动就业1100 余人；新注册成立科技型企业32 家，国家级高新技术企业达到8 家，宝泰隆新材料公司入选“国家技术创新示范企业”；加大人才引进力度，引进硕士研究生51 名。筹集资金4.8 亿元加强经开区、高新区和现代煤化工、石墨烯新材料、生物发酵等专业产业园区建设，园区承载能力和集聚效应明显增强。成功举办石墨烯应用研讨会、冰雪体育旅游文化产业发展论坛、LED 产业模式创新高峰论坛、石墨烯应用创业创新大赛、首届中国绿色产业博览会和第七届中国再生资源产业发展论坛等大型活动，吸引催生了一批新项目落地。

　　坚持绿特色发展方向，现代农业稳步发展。种植结构加快调整，玉米调减16.1 万亩，蔬菜增加10.7 万亩，经济作物达30.8万亩。畜牧业快速发展，形成了一批具有地方特色的畜产品品牌。深入推进农业“三减”行动，农产品监测合格率达96.5%。七台河大米获地理标识认证，绿色有机认证面积达80 万亩。新型经营主体带动作用更加明显，龙头企业带动基地90 万亩、农户3.8 万户。以龙谷农业科技园为代表的设施农业加快发展，7个“互联网+农业”基地通过省级验收。美丽乡村建设不断推进，禹森薰衣草庄园、震东峰生态园、金沙新区葡萄园等休闲观光农业快速发展，形成一批休闲观光引领型、特色产业拉动型、民俗体验型特色村庄。切实保障农村饮水安全，划定112 个农村水源地环境保护区。大力开展农村环境综合整治，完成畜禽养殖限养区、禁养区划定工作，生态乡镇、生态村创建工作深入推进。打好脱贫攻坚战，实施精准扶贫，突出产业扶贫、金融扶贫、光伏扶贫、电商扶贫，4981 户12025 人实现脱贫，40 个贫困村全部实现“三通三有”。

　　加快完善城市功能，城乡环境持续改善。坚持生态为先、建管并重，城市更加秀美宜居。城市供水工程顺利推进，隧洞工程克服地质条件复杂困难实现全线贯通。实施生产生活供水分离工程，新建管线7 公里。完成城区供热主管网、七矿分散供热区域并网改造，改造老旧供热管网9.13 公里，新增供热面积150 万平方米。依宝公路七台河段大修建成通车，牡佳高铁七台河段开工建设。北岸新城长途客运站新建项目、茄子河客运站改造项目基本完成，年底前投入运营。新上线电动公交车85 台，调整新增6 条线路，基本实现城区公交全覆盖。推进建设中心区雨水管网工程，新建中心河污水处理厂，万宝河截污纳管工程完工，生活垃圾发电厂上网发电，医疗废物处置中心实现市场化运营。开展“治霾卫蓝”“治污净水”“保土护田”等14 个环境治理专项行动，淘汰燃煤小锅炉360 台、老旧黄标车1138 台。新建7个微型大气自动监测站，空气质量优良天数301 天。实施仙洞山公园道路改造、城区节点绿化升级、新建学子园等工程，新增绿地10 公顷。全面落实市县乡村四级河长制，编制一河一策方案，完成内河“体检”，万宝河、新老七台河等城市内河污染治理全面启动。倭肯河湿地保护与恢复建设工作扎实推进，桃山湖国家湿地公园通过省级验收，新兴采煤沉陷区综合治理工程获国家试点政策支持。

　　始终坚持发展惠民，社会事业全面进步。克服财政收支矛盾，全力办好民生实事，民生领域支出占财政一般公共预算支出81.5%，比上年提高8 个百分点。完成采煤沉陷区棚户区改造建设和安置任务，实物安置4167 套，货币化安置19056 套，发放购房补贴22.91 亿元。完成3274 套公（廉）租房建设，达到分配入住条件。接收七矿物业移交小区23 处、住宅486 栋，通过改造新建等方式解决1514 户建档贫困户的住房问题，城乡居住环境进一步改善。完成6 个历史遗留问题项目2427 户产权证办理工作，亚太国际商城、金桥家园烂尾楼项目重启建设，252 户居民回迁入住，有效解决涉事百姓回迁难、办证难问题。多渠道促进创业就业，实现新就业8209 人，零就业家庭动态清零。成立龙创再就业服务公司，接收七矿公司第二批分流人员3364 人。发放创业小额担保贷款1900 万元。推进机关事业单位养老保险制度改革，城乡养老、医疗、工伤、生育保险等参保人数继续扩大。调整提高了企业、机关事业单位退休金待遇和困难群众基本生活保障标准。全面完成城乡居民医疗保险整合，城乡居民基本医疗保险实现“六统一”，与部分省市实现异地就医直接结算。推行贫困户“先诊疗、后付费”服务模式，困难群体大病保险起付线降低40%，报销比例增加5 个百分点。全面实施教育惠民项目，加强薄弱学校改造，市中小学综合实践基地、青少年活动中心、六小教学楼基本建成，市、区13 所中小学操场改造工程顺利推进。统筹推进城乡义务教育一体化发展，区县义务教育基本均衡发展全部通过国检，高考本科入段率、重点大学录取率实现较大突破。医疗卫生条件明显改善，市残疾人康复中心投入使用，市人民医院医师规范化培训基地、勃利县人民医院扩建工程、茄子河区医院综合楼主体工程完工，七煤医院综合楼开工建设。公立医院综合改革顺利推进，市民基本公共卫生服务人均标准提高到50 元。实施“百名医科研究生计划”，引进医学硕士14 人。文化惠民工程扎实推进，新建20 个社区文化中心和55 个标准化中心村文体设施，市文化中心主体完工，群众文化活动丰富多彩。“国家短道速滑七台河体育训练基地”获国家批准，成为全国唯一冬季项目单项国家训练基地。我市升格为“国家重点高水平体育后备人才基地”，培养输送的运动员全年获世界级金牌、国家级金牌10 枚。

　　稳步推进各项改革，发展动力活力明显增强。坚持以改革破难题、以改革增活力，以改革促发展。深化“放管服”改革，加快推进“互联网+政务服务”，推进审批职能集中、公共服务事项集中，实行“一窗办理”“一网通办”，市本级行政审批事项精简到260 项，产业项目并联审批时限由109 天大幅压缩至32 天，产业项目审批环节市级行政事业费实行“零收费”。商事登记实现“23 证合一”，全市新注册企业1005 户、个体工商户6542 户，分别增长31.0%、47.0%。积极支持七矿公司解困振兴，七矿公司实现扭亏增盈11 亿元；全面完成七矿公司厂办大集体改革，拨付职工厂改资金9055 万元。积极推进财税改革，加强公共财政体系建设，完成市级预算单位整合。创新投融资体制，设立产业引导资金、新材料产业基金，兑现企业扶持资金5.4 亿元；热力公司资产证券化融资4.2 亿元；化解农信社不良贷款3.5 亿元，农信社改制取得突破性进展；鼓励社会资本参与公共设施建设，市中医康复诊疗中心PPP 项目启动实施。积极推进农业综合改革，在全省率先开展利用保险资金支持“三农”发展试点；农村土地确权外业测量总面积240.8 万亩，勃利县土地确权合库工作完成；4 个省级农村集体产权制度改革试点村完成股权量化阶段性工作。开展城市管理综合执法体制改革，建立市一级“大城管”管理体制，解决市、区城市管理责权不等、职责不清等问题。坚决守好安全底线，社会保持和谐稳定。深入贯彻安全发展理念，创新社会治理方式，安全生产形势和社会治安总体稳定。把推进兼并重组作为煤矿安全发展的治本之策，推动130个地方煤矿组建证照统一、产权明晰、统一经营的15 家实体煤矿企业集团，为地方煤矿可持续发展、安全发展打下坚实基础。制定出台《地方煤矿安全生产监督管理暂行办法》，强化“人防”“技防”“制防”煤矿安全综合监管措施，建立煤炭行业安监人员交流轮岗、专家安全评估、尽职免责等制度，采取无人机等现代化信息手段全时段监控关停矿井。坚持对消防森防、危险化学品、道路交通、建筑施工、特种设备、易燃易爆品、人员密集场所等重点领域高压严管，全年未发生较大安全事故。开展民办养老机构安全隐患整治，关停19 家无证运营机构。坚持“四个最严”，加大食品药品监管力度，确保百姓“舌尖上的安全”。全面启动“七五”普法，深入推进公安“六大工程”建设，开展“三打击一整治”等专项行动，“9·8”特大地下钱庄案侦办取得重大成果，治安案件同比下降32%，人民群众安全感进一步提升。坚持领导接访制度，实行带案下访、包案化解，集中开展信访积案清理、非访和越级访治理、秩序维护三个专项会战，有效化解一大批疑难信访问题。

　　严格推进依法行政，政府建设不断加强。按照职权法定要求,对市本级行政权力清单和责任清单进行规范调整，清单之外再无权力。健全依法决策机制，深入推行法律顾问和公职律师制度，全面推行行政执法“双随机、一公开”工作机制，依法行政取得新进展。强化效率意识，政务服务窗口单位实行周末延时服务。对行政审批、行政权力、公共服务事项进行流程再造，解决环节多、周期长、效率低等问题，为民施政取得新成绩。围绕市九次党代会确定的重点工作、市十一届人大一次会议各项决议和代表意见建议，针对群众关注的热点难点问题，强化督办落实，代表建议办理满意和基本满意率达100%，务实勤政取得新突破。全面加强政府系统党的建设，推进“两学一做”学习教育活动制度化常态化，坚持不懈纠正“四风”，扎实开展作风整顿，给予党政纪处分39 人；坚持零容忍态度惩治腐败，给予党政纪处分400 人。加强审计监督，查出违规问题涉及金额1631 万元，从严治政取得新成效。

　　各位代表，成绩来之不易。这是省委省政府和市委统揽全局、正确领导的结果，是市人大、市政协有力监督、大力支持的结果，是各级各部门和全市人民团结拼搏、共同奋斗的结果。在此，我谨代表市人民政府，向全市广大干部群众，向关心支持我市发展建设的各界友人，表示衷心的感谢和崇高的敬意！

　　在肯定成绩的同时，还要看到我市发展不平衡不充分的问题还很突出，满足人民日益增长的美好生活需要还有不少短板和弱项。主要是：替代产业支撑能力不足，战略性新兴产业培育发展不快，科技创新能力偏弱，资源环境约束加剧；一些重点领域和关键环节的改革不到位，市场化程度低，机制不完善，创新人才偏少，微观市场主体活力不足；城乡区域发展不均衡，民生领域仍有不少欠账，脱贫攻坚任务艰巨，安全生产基础不牢；城市基础设施、公共服务设施不完善，城市经营管理水平亟待提升；少数政府部门和工作人员主动服务、勇于担当意识不强，部分干部精神不振、能力不足，思想观念和工作作风亟需转变。有效破解这些问题，如期完成全面建成小康社会的各项目标任务，仍需付出更大的努力。我们必须以时不我待、只争朝夕的精神，加快推动转型发展、实现全面振兴，与全国全省人民同步全面建成小康社会。

　　二、2018年重点工作

　　2018 年是全面贯彻落实党的十九大新战略新部署的开局之年，是决胜全面建成小康社会的关键之年，也是推进供给侧结构性改革的深化之年，更是我们走进新时代贯彻新思想踏上新征程大有作为的一年。做好2018 年政府工作总的要求是：以习近平新时代中国特色社会主义思想为指导，全面贯彻落实十九大精神和习近平总书记对我省重要讲话精神、省十二次党代会和省委十二届二次全会精神，紧紧围绕新时代中国特色社会主义发展的战略安排和省委建设现代化新龙江的战略部署，坚持新发展理念，按照高质量发展要求，统筹推进“五位一体”总体布局、协调推进“四个全面”战略布局，按照市九次党代会、市委九届二次全会工作部署，坚持稳中求进工作总基调，以转型发展为主线，以改革创新为动力，以增进民生福祉为目的，以生态文明建设为前提，以安全发展为基础，突出抓重点、补短板、强弱项，创新实施“七大战略”，坚决打好“六大攻坚战”，努力实现“五个新突破”，推动质量变革、效率变革、动力变革，不断激发内生动力和发展活力，全面筑牢转型发展全面振兴的坚实基础。经济社会发展的主要目标是：地区生产总值增长6.5%以上，规模以上工业增加值增长5.0%左右；全社会固定资产投资增长10.0%，财政一般公共预算收入按可比口径增长5.2%；城镇居民人均可支配收入增长7.1%，农村居民人均可支配收入增长7.8%；城镇登记失业率控制在4.5%以内；万元GDP 能耗下降3.5%，万元GDP 二氧化碳排量下降3.5%。

　　（一）发展壮大实体经济，提高经济发展质量效益

　　推动煤城转型振兴，出路是发展民营经济，关键是抓好产业项目建设，核心是推动创新升级。要坚持“增量崛起”与“存量变革”并举，打好转方式调结构攻坚战，通过要素推动、投资拉动、创新驱动，不断延长产业链、嵌入创新链、提升价值链，加快民营经济成长壮大，加快六大产业做大做强，加快实体经济全面振兴。

　　大力发展民营经济。深入贯彻落实市委“在推动民营经济发展上实现新突破”的要求，进一步激活民营经济立市的优秀市场意识基因，加快推进东北地区民营经济试点市建设。创新政策支持体系，出台促进民营经济加快发展、金融支持实体经济、促进创业创新、鼓励投资促进产业项目建设等“黄金10 条”系列政策措施，以政策创新助推民营经济发展。下大力气解决民营经济发展“玻璃门、弹簧门、旋转门”问题，全方位营造尊商、重商、安商、富商社会氛围。大力支持创新创业，打造众创、众包、众扶、众筹平台，鼓励更多社会主体投身创新创业。开展企业家成长专项行动，加大民营企业家培训力度，激发和保护企业家精神，充分释放企业家才能。深入实施小微企业成长工程，打造一批专、精、特小微企业集群，鼓励小微企业与大企业协作配套，推动更多小微企业实现个转企、小升规。大力支持规模以上民营企业做大做强，通过规改股、股上市，不断取得更好更快发展。

　　抓好产业项目建设。编制实施产业攻坚三年行动计划，现代煤化工产业基地、新材料新能源基地、黑龙江东部新型商贸物流中心建设规划，完善产业发展战略布局和项目支撑体系。坚持“四帮四保”项目推进机制，重点推进千万元以上产业项目38个，总投资197 亿元。加快建设现代煤化工产业基地。进一步完善煤化工园区基础设施，确保宝泰隆稳定轻烃、隆鹏清洁化学品等在建煤化工大项目建成投产。推动隆鹏与北京三聚深化合作，谋划建设尿素制10 万吨三聚氰胺项目。与七矿公司建立长协煤供应机制，提高大型煤化工企业产能利用率。大力发展石墨新材料、生物发酵产业，在培育替代支柱产业上实现新突破。以电池负极材料、石墨烯应用产业为重点，加快新材料产业基地建设。推进万锂泰5 万吨石墨烯锂电池负极材料产业集群、宁波金墨2000 吨石墨烯多元磷酸盐正极材料、金羽环保钛电池等项目建设。依托鑫科纳米院士工作站、宝泰隆石墨烯研究院技术优势，继续举办石墨烯应用创新创业大赛，吸引集聚更多石墨烯应用产业项目落地发展。加快生物发酵产业园区供水管线、污水处理厂、电厂等基础设施建设，大力发展生物和医药产业。加快推进中瑯生物年产5 万吨衣康酸10 万吨葡萄糖酸钠项目建设，争取哈药总厂原料药项目落地开工。依托哈宜公司环保技术优势和污水处理低成本优势，加大新项目引进力度，尽快把生物和医药产业打造成支柱产业。加快发展传统优势产业。立足我市绿特色种植养殖业发展基础，加快金晖食品方便米饭、伊鑫清真食品、利健科技养生产品等项目建设，壮大绿色食品产业规模。引导农业机械、矿山机械、医疗器械、节能装备等制造业企业与外来资本、先进技术和市场需求对接，积极引进新技术、开发新产品。推进双叶家具生产线智能制造、柔性制造升级改造项目投产达效，扩大个性化定制，打造家具行业智慧工厂。帮助升华医疗器械做好GMP 认证，促进项目尽快建成投产。按照“一区多园”模式，推进七台河高新区建设，深化园区管理体制改革，落实各项扶持政策，促进高技术产业集聚发展，争创国家石墨烯新材料、电池负极材料特色产业基地。继续加大再生资源回收利用产业园区、金沙健康产业园区、勃利开发区等园区基础设施建设力度，提升产业承载能力。

　　努力提高全要素生产率。推动七矿公司学习皖鲁经验深化改革，建立内部市场化机制，提高资源配置效率，尽快实现全面解困振兴。加强与省地矿局合作，实施“找矿行动”，加大资源勘查开发力度。全面完成地方煤矿实质性整合重组，推进地方煤矿扩能改造，有效释放安全产能。以降成本为核心，深入推进“三去一降一补”，加大减税清费力度；加快淘汰落后产能，依法关闭浪费资源、污染环境企业；加强“僵尸企业”“半截项目”“停产半停产企业”分类处置，有效盘活闲置产能。支持电力调度控制中心项目建设，全力保障电力安全可靠供应，推进适合条件的大用户直接购电，实施用电大户新增电量电价补贴制度，降低企业用电成本。积极开展金融创新，激励金融机构争授信、增信贷，提高金融服务实体经济效率。大力引进和培育发展地方金融机构，完成市农信社改制组建农商行。鼓励债权、股权、基金等融资方式，支持企业进入资本市场筹集资金，推动鹿山集团等企业上市（挂牌）融资。

　　加大科技创新力度。在提升各类人才素质上实现新突破，充分发挥人才在科技创新中的引领作用。围绕转型振兴大力引进“千人计划”等各类高科技人才，加快人才公寓建设，更好地吸引人才、留住人才。推动产学研用结合，加快鑫科纳米院士工作站建设，深化与清华、哈工大、东北农大、哈体院等大专院校合作，组织开展“七台河专家行”活动，推动高校与企业在产业发展、技术支持、人才培养等方面深入对接。推进开发区“创业苗圃+科技孵化+孵化加速+共享工厂”四位一体的孵化平台建设，促进资本与技术合作、技术与生产合作，形成产业创新集群。用好产业引导资金、新材料产业基金，加大对战略性新兴产业、高新技术产业项目扶持力度，培育打造一批科技创新型企业。

　　（二）抓好农业调结构转方式，加快推进农业现代化

　　把增加绿色优质农产品供给放在突出位置，大力推进农业供给侧结构性改革，促进农村一二三产业融合发展，构建现代农业产业体系、生产体系、经营体系，促进农业增效、农民增收。

　　调整优化农业产业结构。实施优质粮食工程，发展优质高效粮食生产，确保粮食安全。引导农民适应市场需求，进一步优化种植结构，粮经种植比由89：11 调整到85：15。加快发展高钙富硒水稻、高蛋白食用大豆，扩大青贮玉米，适当发展鲜食玉米。因地制宜发展红小豆、芽豆、红谷子等杂粮杂豆，高钙菜、白瓜子、月苋草等特色经济作物，蓝靛果、红松果、大榛子、山野菜、黑木耳等林下产业。大力发展设施农业，实施“菜篮子惠民行动”，扩大棚室和露地蔬菜生产，淡季蔬菜自给率提高到60%以上。积极落实省委、省政府“两牛一猪”规模标准化基地、畜牧贷等扶持政策，大力发展标准化规模养殖、绿特色养殖，做大做强肉牛、肉羊养殖，引进高端肉牛养殖项目，推进乌驴养殖项目。深入实施“三减两增一提高”行动，加强粮食质量监测体系、农产品质量监测体系建设，加大“三品一标”创建力度，提升农产品供给质量和效益。

　　推进农村一二三产业融合发展。贯彻落实“粮头食尾、农头工尾”，推动农林牧渔结合、种养加销一体，壮大农畜产品精深加工产业。推进多种形式土地适度规模经营，培育壮大新型农业经营主体，深入开展联合社、规范社、示范社创建活动，鼓励发展家庭农场、种养大户。健全农业社会化服务体系，与东北农大、东北林大、湖南大学等开展农业科技合作，建设现代农业产业园、科技园、创业园。加大新型职业农民培养力度，加快建设知识型、技能型、创新型新农民队伍，推动农民创业。持续抓好省级“互联网+农业”示范基地建设，鼓励扩大基于互联网和新商业模式下的展示营销、定制营销等农产品销售方式，加大绿特色农产品在“七台河大米网”等电商、微商平台线上销售力度，拓宽渠道，创建品牌，真正实现“种得好，卖得更好”。鼓励发展田园体验小镇、农林体验小镇等新型融合发展模式，推进龙谷科技园、禹森薰衣草庄园、康草堂北药基地等集农业、观光、加工及物流于一体的综合项目加快发展。推动龙谷科技园争创省、国家农业科技示范园区。

　　坚决打赢脱贫攻坚战。坚持扶贫与扶志、扶智相结合，继续推进产业扶贫、金融扶贫、光伏扶贫、电商扶贫，因村因户因人施策，建立贫困户脱贫长效机制。按照“两不愁”“三保障”标准，逐项对照教育、医疗、住房和饮水安全保障情况，集中力量补齐短板。严把贫困发生率、漏评率、错退率和群众认可度等脱贫评价指标，落实精准退出制度，做好勃利县脱贫摘帽工作。

　　（三）加快培育新业态新模式，大力发展现代服务业加快培育新产业、新业态、新商业模式，推动商贸、文化、体育、旅游等产业协调融合发展，建设龙江东部新型商贸物流中心。

　　巩固壮大商贸物流业。加快推进传化智慧物流、中国供销商贸博览城二期、海生新零售小镇项目建设，积极引进大型现代物流企业，发展壮大公路货运枢纽，加速形成商贸物流集聚效应。推动新玛特、东北亚等传统零售业增强消费体验，加快线上线下融合发展，实施七彩城、步行街整体改造，打造智慧商圈。加快培育共享经济、总部经济等新业态和新商业模式。

　　打造“龙江东部电商谷”。优化电子商务产业园区运营和管理方式，整合现有资源，提升电商园、创客空间承载和服务能力，积极引进第三方支付平台落户我市。做大做强“七台河电商谷”等自主平台，培育电商领域龙头企业和拳头产品。借助双叶家具销售网点，在全国主要城市设立七台河特色产品店，扩大地方产品线上线下销售覆盖范围。加强互联网创业和营销培训，扩大电商、微商数量和规模。

　　加快发展数字经济。推动大数据、智能化、移动互联网、云计算和物联网等新一代信息技术同实体经济深度融合，引导企业应用信息数据技术开展技术创新、产品创新、管理创新和商业模式创新。推进数据资源整合和开放共享，加快教育、就业、社保、卫生、交通、公安、电子政务、智慧城市等领域大数据普及应用。加快光纤网、5G 移动通信网、无线局域网等信息基础网络建设，提高对“大智移云物”产业支撑能力。引进华为科技云计算、偶然网络大数据项目，谋划建设“互联网产业园”，全力推动大数据产业。

　　积极发展冰雪旅游体育文化产业。深度挖掘城市历史文化，依托市文化中心设置抗联红色文化馆、冠军文化馆等展馆，辟建劳模文化馆。推动文化产业加快发展，创作一批以抗联文化、冠军文化、矿工文化、创业文化为题材的文艺精品，举办冰雪文化产业论坛，推出《冰之梦》等大型演艺活动，讲好七台河故事，在提升文化软实力上实现新突破。发挥“国家短道速滑七台河体育训练基地”作用，争取承办短道速滑等高水平赛事活动，逐步把“中俄青少年短道速滑联赛”打造成具有国际国内影响力的品牌赛事。开展“冰雪嘉年华”“赏冰乐雪”系列活动，提供丰富的冬季冰雪旅游产品，推动全民参与冰雪运动，增强参与性和娱乐性，形成专业赛事、休闲娱乐为一体的冰雪旅游产业格局。推进红色旅游、冰雪旅游同森林旅游、乡村旅游、休闲旅游相结合，保护性开发兰棒山抗联第二路军总部（原吉东省委）遗址、勃利密塞爱国主义教育基地等景区，建设周保中将军纪念馆，改扩建烈士纪念馆，打造红色旅游基地。推进石龙山、西大圈国家森林公园景区、吉兴湖景区升级改造及休闲、康养等旅游项目开发，增加优质旅游产品供给。

　　（四）加快构建外向型经济体系，深化和扩大对外开放

　　以对外通道建设为突破口，积极对接“中蒙俄经济走廊”建设，打破处于全省物流运输体系“盲端”的发展瓶颈，完善发展战略布局，多维度推进全面开放，不断拓展对外开放领域，深化对外开放层次。

　　加快对外交通体系建设。抓好牡佳高铁站区规划建设，谋划争取通用机场立项，建设综合交通物流枢纽。积极对接同江铁路口岸，谋划建设七宝铁路，加速融入中俄跨境物流运输体系。实施七密公路改扩建工程、七勃铁路道口平改立工程，开工建设七桦路北出口、开发区西出口道路，规划建设金沙新区至桦南森工一级道路，形成对外开放快速通道体系。

　　推动形成区域协调发展格局。加强与龙江东部城市群、东北东部城市群战略合作，创新区域间的合作模式，推动与周边城市产业一体化、交通一体化、市场一体化合作，建立多层次、多范围、多样化的互补合作机制，实现互利互惠合作共赢。推进建设宝泰隆石墨烯小镇、海生新零售小镇、新兴双创小镇、冰雪体育小镇、龙谷田园体验小镇、兰棒山红色文化小镇、石龙山康养小镇等一批对周边地市具有辐射带动能力的特色小镇，实现城市功能、产业在更大范围内合理布局，促进人流物流集聚、生产要素集聚。

　　大力发展外向型经济。利用俄罗斯远东开发有利时机，支持企业积极稳妥“走出去”，在矿产资源开发、森林资源开发、农产品基地建设、再生资源回收利用等方面积极寻求合作，打造跨境产业链。完善海关监管场所报关报检条件，为企业开展进出口业务提供便利化服务；积极发展电子口岸、跨境电商等内陆经济开放新模式。深化与江门等发达地区对接合作，更多地“请进来”，在珠三角、长三角、京津冀等地区建立七台河商会，加大城市整体性宣传、推介、营销力度，寻求互补合作的市场机会，注重引资与引智并举，开展专业招商、靶向招商、飞地招商，通过各种形式引进先进理念、高新技术、战略投资、成熟模式。

　　（五）提升城乡建设管理水平，打造山青水绿宜居家园坚持精心规划、精致建设、精细管理，强化城市承载与服务等功能，提升城市品位，为发展提供硬件支撑、为百姓打造宜居环境、为后代守护美丽家园。

　　完善城市规划体系。以规划引领城市建设和发展，编制实施综合交通运输体系、商业网点布局、海绵城市、智慧城市建设、生态环境保护和修复治理等规划。推进“多规合一”，编制统一的空间规划，科学划定“三区三线”，合理安排城乡、产业和基础设施布局。谋划建设城市规划展览馆，让市民认知规划、支持规划，自觉参与城市建设和管理。

　　完善城市路网建设。实施人才街、大同街东延、文华路北延、景丰路南延等道路建设和改造工程，打通断头路、开放小区路，扩大道路容量，畅通城市路网。继续推进公交车更新换代，建设北岸新城新能源公交场站，推进七勃客运公交化运行，开辟市区至依兰高铁站快速客运专线，为百姓提供便捷出行条件。

　　统筹推进城市管网建设。加快推进城市供水工程，确保如期实现通水。实施净水厂水源互通、供排水总公司水厂、胜科水厂升级改造和供水计量设施改造工程，新建茄子河、金沙新区供水加压泵站，推进供水主干线、分支及小区内部管网改造，加强二次供水管理，保障供水水质和供水安全。完成分散供热区域集中供热并网后续工程。新建宏伟镇污水处理厂，推进实施中心城区污水管网工程，改造七星花园、欣源小区等地截洪排水设施，推进北岸新城、东方花园等易涝区域专项整治。

　　加强城市绿化美化亮化。做足滨水文章，与倭肯河治理工程相结合，综合地形地貌高标准规划设计，建设桃山湖坝下倭肯河湿地公园。实施仙洞山公园、人民公园、应龙山公园、万宝公园及坝下玫瑰园、荷花园、垂钓园升级改造工程，精品化推进公园绿化、街路节点绿化、居住小区绿化。实施电视发射塔和新建楼体亮化工程，修复老旧亮化设施，保证亮化完好率亮灯率。

　　提升城市管理水平。强力开展“市容环境专项整治行动”，实施违法建筑、早市夜市、露天烧烤、噪声污染等专项整治，提升城市整体形象。规划建设文明夜市一条街，完善奋斗路早市配套设施。加强城市公共空间管理，合理规划建设绿地、公厕、停车位等设施。提高环卫工人工资待遇，推动环卫保洁服务市场化，提高环卫“四清”机械化作业率。深入推进城市管理综合执法改革，完善“大城管”体系，推进数字化城管建设。

　　打好污染防治攻坚战。坚持全民共治、源头防治，加大环境治理力度。着力解决突出环境问题，持续开展大气、水、土地污染防治行动，全面实施“工业污染源达标排放计划”。加快推进万宝河及新老七台河等黑臭水体治理。新建危废处理中心，实施垃圾填埋场坝体加固工程和渗滤液处理工程，新建生活垃圾转运站，推动建立城乡一体化生活垃圾收运体系，逐步开展垃圾分类回收。

　　保护修复生态环境。实行最严格的生态环境保护制度，统筹山水林田湖草系统治理。加快推进桃山湖国家湿地公园、那丹哈达岭地质公园建设。落实河长制、湖长制，强化水源地生态保护，推进倭肯河流域一体化治理，开展违建、污染、河道采砂等清河专项行动，推进沿河退耕还湿和岸线生态保护。持续开展保土护田行动，加强农业面源污染防治。实施矸石山、沉陷区生态恢复治理，大力开展造林绿化，建设采沉区天然林储备工程。

　　加快美丽乡村建设。坚持农村产业发展与新型城镇化相结合，推动公共服务向农村延伸，加快城乡一体化发展。推进农村硬化、净化、亮化、绿化、美化，实现农村通路、通电、通自来水、通宽带网全覆盖。开展“农村人居环境整治行动”，推进农村垃圾集中清运、秸秆综合利用、畜禽粪便无害化处理和资源化利用。积极推进“厕所革命”，加快改水、改灶、改圈，建设产业兴旺、生态宜居、乡风文明、治理有效、生活富裕的和谐美丽新农村。

　　（六）切实保障和改善民生，不断提高市民幸福指数

　　坚持把人民对美好生活的向往作为奋斗目标，问计于民、问需于民，尽力而为、量力而行，扎实办好民生实事，推动解困型民生向幸福型民生转变，在保障基本民生上实现新突破，确保发展成果更好更多惠及全市人民。

　　抓好保障性安居工程建设。推进棚户区改造由城镇棚户区向城市危旧房、城中村及非集中连片棚户区延伸，计划改造2977 户。实施七矿移交老旧房屋维修改造，综合改造老旧单散危楼和小区。全面实施物业管理星级评价、末位淘汰制度，提升物业服务水平。继续推进农村危房改造，改善农村居住条件。

　　推进教育全面均衡发展。完成职业学院附属幼儿园、杏树中心幼儿园等城乡幼儿园改造项目，加大对民办幼儿园的支持和管理力度，提高普惠型幼儿园幼儿在园比例。提高义务教育均衡发展水平，新建3 所中小学校，缓解大班额问题。建设岚峰学校教学楼等项目，改善乡镇学校办学条件。全面完成中小学校操场改造工程。加快推进教育信息化，提高信息化网络环境和装备水平。推进专家型校长队伍建设工程，解决偏远学校专任教师短缺和待遇问题。加强师德师风建设及教师培训工作，建设“四有好教师”队伍。加强社会主义核心价值观和中华优秀传统文化教育，强化德育教育、素质教育。加强学校体育工作，继续办好体育特色校，夯实体育后备人才基础。实施高中普及攻坚计划，深化普通高中课程体系、教育教学改革，不断提高高中阶段整体办学水平。树立现代职业教育办学理念，坚持“实用教育”理念和“校企双制”的培养模式，提高职业教育办学水平和毕业生就业率。积极推进七台河公共实训基地建设，加快建设职业技师学院创业大厦和实验楼工程。

　　提升城乡居民医疗健康水平。推进市人民医院改扩建、七煤医院综合楼、市中医院中医诊疗康复中心和“治未病”中心项目如期建成并投入使用。加快卫生信息化建设，开展远程智慧医疗。实施重点疾病预防控制计划、三级医疗网络平台建设计划、健康科技创新行动计划、专业人才引进和培养计划、专科医联体建设计划，切实提高医疗服务质量。开展“三减三健”行动，以倡导健康观念、普及健康生活、优化健康服务、完善健康保障、建设健康环境、发展健康产业为重点，打造“健康龙江”示范市。推进医养结合，加快天赐智慧医养中心项目建设，扶持合格民办养老服务机构健康发展，推进公办养老机构公建民营改革。加强社区日间照料中心、儿童之家、养老服务站、红十字服务站等建设，提升社区养老、托幼、救助服务能力。

　　加强社会保障体系建设。提高统筹层次、基金支撑能力和待遇水平，完善经办管理服务体系，全面完成全民参保计划，实现社会保障法定人员全覆盖。完善城乡统一的居民基本医疗制度，推行按病种付费，全面实现异地就医直接结算。建立健全各层次大病保险制度，切实减轻参保患者大病医疗负担。完善城镇职工基本养老保险和城乡居民基本养老保险制度，有序推进机关事业单位基本养老保险制度改革。更多关注扶助社会弱势群体，统筹社会救助体系建设，完善最低生活保障制度，确保低保救助对困难群体全覆盖。积极发展社会福利、人道救助、慈善和残疾人事业，保障困难群众和弱势群体生活。

　　促进创业带动就业。通过盘活闲置资源建设创业园、创业基地、育成中心、科技企业孵化器等创业载体。设立创业创新专项扶持资金，完善促进创业的政策支持体系，打造“零成本”创业环境。加大“电子就业地图”建设，抓好大学生、农民工、城镇转移职工、就业困难群体就业工作，形成统一的人力资源市场和城乡均等的公共就业服务体系，有效保障就业权益，实现比较充分就业。

　　（七）提升社会治理能力，切实维护社会和谐稳定

　　牢固树立安全发展理念，积极推进系统治理、依法治理、综合治理、源头治理，提高社会治理水平，维护社会公平正义，确保人民安居乐业，社会安定有序。

　　全力抓好安全生产工作。坚持生命至上，安全第一。大力推进安全生产领域改革创新，压紧压实各行业领域、各级各部门安全生产责任特别是企业主体责任，切实增强安全事故防范治理能力。实施安全生产综合治理，抓好重大危险源管控和各项管控制度的建立和落实。突出抓好煤矿安全生产，严防违法违规、超能力和不安全生产。推进安全生产大检查和专项整治常态化，采取多部门联合执法，持续开展煤矿、危险化学品、道路交通、建筑工地、消防森防、特种设备、城镇燃气、人员密集场所等行业领域安全生产大检查和专项整治，及时消除安全隐患。加快东部煤化工应急救援基地建设，继续加强公安消防和矿山救护救援能力建设。强化食品药品安全监管，争创“食品安全城”。

　　提升社会安全保障能力。继续推进公安“六大工程”建设，提高对各类风险发现、防范、化解、管控能力。加强社会治安防控体系建设，建设“雪亮工程”，织密源头防控、重点防控、科技防控、区域防控和网络防控相结合的治安防控网。依法严厉打击危害公共安全、扰乱生产经营等各类违法犯罪，扎实推进更高水平的“平安七台河”建设。

　　做好维护社会稳定工作。扎实做好源头治理和教育稳控等工作，狠抓矛盾纠纷排查化解。加强和完善城乡社区治理，合理配置社区用房，拓展社区服务功能，深化网格化管理，争创“全国社区治理和服务创新实验区”。全面落实诉讼和信访分离制度，建立部门信访职责清单，健全完善律师参与信访工作、依法维权和化解纠纷机制，集中开展积案化解、重信重访治理、信访业务规范等专项行动，扎实推进依法分类处理信访诉求，切实维护群众合法权益。

　　（八）打好重点领域改革攻坚战，增强经济社会发展活力

　　准确把握习近平新时代中国特色社会主义思想蕴含的改革精神、改革部署、改革要求，突出抓好有利于经济发展、有利于惠及群众、有利于社会稳定的重点领域和关键环节改革，充分释放发展活力。

　　深化市场化改革。坚持非禁即入，加大力度向市场、社会放权，构建“亲”“清”政商关系、“和”“融”民商关系，保护企业家权益，打造最适宜经济发展的公平法治环境。深化行政审批制度改革、商事制度改革，全面实施市场准入负面清单制度，加快推行企业投资项目承诺制。进一步推进市、区事权与支出责任划分改革，深化政府采购、财政投资评审管理改革，推动绩效预算改革全覆盖。推进公用事业领域改革，组建水务集团、公交集团。坚持“专业事专业部门做”，建立政府投资项目统一建设、管理、服务机构。鼓励社会资本参与市政设施、公共服务等项目建设，扩大政府购买服务范围。进一步完善公共交易平台建设，规范公共资源交易活动，提高市场化招标质量效率。推进农业综合改革。加快农村土地承包经营权确权登记颁证，研究制定“三权分置”实施意见，形成农户、村集体和经营主体共享土地权利的合理机制。复制试点村改革的成功经验，深化农村集体产权制度改革。积极推进农业转移人口市民化改革。

　　完善文教卫生体制改革。全面推进公立医院综合改革，加快建设现代医院管理制度，进一步抓好家庭医生签约服务和紧密型医联体建设，完善分级诊疗制度。统筹义务教育人才资源均衡配置，启动校长教师交流改革试点工作。实施市直属小学学生学业水平评价制度改革，推进城乡义务教育一体化改革。

　　抓好其它专项改革。建立健全质量品牌奖励机制，设立“市政府质量奖”，提升产品质量、服务质量。强化社会诚信体系建设，推行“红黑名单”管理制度，建立失信者联合惩戒机制。稳步推进国有林场改革，建立森林资源资产管理制度。

　　三、加强政府自身建设

　　新时代要有新气象、新作为。煤城转型时不我待，我们要以更加进取的精神状态、更加务实的责任担当、更加执着的为民情怀，胸怀梦想，夙夜在公，激情工作，全面提升政府执行力、凝聚力和战斗力。

　　创新施政理念，提升服务效率。坚持问题导向、效率导向、公平导向、目标导向，深入推进简政放权，加快转变政府职能，进一步放宽准入、精减事项、再造流程、优化服务，推动政府管理由事前审批向事中事后监管转变。建设市民服务中心，把应纳入的市本级审批和服务部门全部纳入，提供全覆盖、全方位的“一站式”服务。建成覆盖全市、市区乡（社区）联动、合理统筹、一网办理的“互联网+政务”体系，全面推进审批事项、公共权力、公共服务事项网上办理，让网络数据多跑路，让人民群众少跑腿。建立“微市长”“微区（县）长”“微局长”“微警长”24 小时在线微信公众服务平台，第一时间解决企业和百姓诉求，努力打造群众最满意、企业最满意的政府。

　　落实依法行政，提升治理水平。坚持权责法定思维，带头尊法学法守法用法，推行政府法律顾问制度，把法治理念贯穿政府工作全过程，自觉接受人大依法监督、政协民主监督，主动接受社会和舆论监督。加强新型智库建设，建立健全决策咨询制度，推进重大决策科学化、民主化、法制化。继续深化政务公开，落实新闻发言人制度，充分利用微博、微信等新媒体，及时传递政务信息，及时回应社会关切。深化行政执法体制改革，大力推进基层行政执法规范化，完善行政执法监督制度。

　　打造过硬队伍，提升履职能力。按照政治过硬、本领高强要求，全面打造一支胸有全局、心有规矩、肩有担当、业有专攻的干部队伍，增强学习本领、政治领导本领、改革创新本领、科学发展本领、依法执政本领、群众工作本领、狠抓落实本领、驾驭风险本领，提高研究解决前沿问题、专业问题和具体问题的能力。健全和完善政府多元化用人机制，根据需要探索实行契约化管理的政府雇员制度。坚持尽职免责、失职追责，完善激励约束机制，重点整治政府系统干部队伍在思想观念、工作标准、办事效率、责任担当、工作落实、执行纪律上的突出问题。

　　强化从严治政，提升政府形象。牢固树立“四个意识”，切实增强“四个自信”，坚定不移向以习近平同志为核心的党中央看齐。全面落实从严治党各项要求，弛而不息纠正“四风”，深入开展“不忘初心、牢记使命”主题教育活动。严格落实党风廉政建设责任，持续开展争当“三个好把式”，形成“五个好作风”活动，践行“四零服务承诺”，严厉查处破坏发展环境的人和事，坚决打赢整顿作风优化发展环境攻坚战。加大重大政策措施落实情况跟踪审计，突出公共财政审计监督，严控“三公”经费支出，切实把有限的资金用在发展经济和改善民生上，用政府的“紧日子”换取群众和企业的“好日子”。

　　各位代表，新时代昭示新使命，新使命需要新担当。面对全面建成小康社会的宏伟目标，面对人民群众对煤城转型振兴的美好期待，让我们更加紧密地团结在以习近平同志为核心的党中央周围，以习近平新时代中国特色社会主义思想为指引，不忘初心，牢记使命，在市委的坚强领导下，凝心聚力，开拓进取，奋力谱写煤城转型振兴的历史新篇章！
